# Supplementary material for: Effect of High Hydrostatic Pressure on the Metabolite Profile of Striped Prawn (Melicertus kerathurus) during Chilled Storage
Source: Foods. 2022 Nov 17;11(22):3677. doi: 10.3390/foods11223677 (PMC9689486; doi:10.3390/foods11223677)
Supplement: Supplementary file 1 [file foods-11-03677-s001.zip › foods-2003711-supplementary.pdf]

Effect of high hydrostatic pressure (HHP) on metabolic profiling of striped prawn (Melicertus kerathurus) during chilled storage

Supplemental material

Table S1. Metabolites of striped prawn samples untreated or treated with HHP of 400, 500 and 600 MPa during chilled storage.

		Control			400 MPa				
		day 1	day 6	day 9	day 1	day 6	day 9	day 14	day 21
Amines	Cadaverine	-	-	0.27±0.03	-	-	-	-	-
	Choline	0.41±0.01	0.32±0.07	0.70±0.01	0.43±0.04	0.34±0.07	0.39±0.03	0.48±0.01	0.40±0.02
	Dimethylamine	0.02±0.01	0.03±0.01	0.02±0.01	0.03±0.01	0.01±0.01	0.02±0.01	0.02±0.01	0.02±0.01
	O-Phosphocholine	0.19±0.01	0.2±0.01	0.24±0.01	0.24±0.01	0.19±0.01	0.25±0.02	0.23±0.01	0.18±0.01
	Putrescine	-	-	11.57±1.53	-	-	0.06±0.06	-	-
	sn-Glycero-3-phosphocholine	0.26±0.03	0.28±0.04	0.13±0.01	0.28±0.02	0.21±0.01	0.28±0.01	0.32±0.01	0.34±0.01
	Taurine	19.17±0.21	19.71±1.00	21.21±0.88	20.3±0.27	16.23±0.12	16.31±1.05	15.47±1.67	15.68±0.45
	TMAO	18.35±0.42	23.78±0.09	2.33±0.32	24.41±1.88	18.87±0.55	18.1±0.65	20.83±0.71	16.75±0.61
	TMA	0.08±0.01	0.18±0.03	23.53±0.15	0.12±0.01	0.12±0.01	0.43±0.18	0.31±0.08	0.16±0.01
	Trigonelline	0.14±0.01	0.16±0.01	0.16±0.01	0.23±0.02	0.15±0.01	0.14±0.01	0.15±0.01	0.11±0.01
	Tyramine	-	-	1.03±0.09	-	-	-	0.05±0.01	0.60±0.03
	Uracil	0.20±0.01	0.15±0.01	0.38±0.02	0.15±0.01	0.12±0.01	0.17±0.02	0.16±0.01	0.17±0.01
Amino Acids	Alanine	12.67±0.39	12.45±0.14	16.84±0.12	14.28±0.21	16.12±0.40	15.49±0.60	14.47±0.16	16.00±0.44
	Asparagine	1.81±0.17	1.87±0.06	0.43±0.02	1.72±0.08	2.23±0.06	1.58±0.04	1.77±0.10	2.03±0.02
	Arginine	12.89±1.37	11.93±0.77	6.00±1.13	13.49±0.32	10.37±0.77	11.76±0.15	10.36±0.74	9.23±1.19
	Beta-Alanine	0.16±0.02	0.18±0.01	0.18±0.01	0.15±0.01	0.17±0.01	0.16±0.01	0.14±0.01	0.15±0.01
	Betaine	58.30±0.77	65.42±0.17	61.18±0.71	62.96±0.95	50.77±0.01	53.13±0.17	52.36±0.39	51.93±1.86
	Creatine	0.41±0.17	0.17±0.09	0.77±0.01	0.18±0.03	0.05±0.02	0.31±0.19	0.12±0.01	0.22±0.14
	Glutamate	2.23±0.30	2.23±0.20	6.38±1.40	2.41±0.98	3.45±0.22	2.51±0.38	2.50±0.11	5.34±0.27
	Glutamine	6.47±0.18	7.80±0.21	5.10±0.46	5.51±0.53	6.47±0.04	5.59±0.12	5.54±0.09	5.84±0.27
	Glycine	65.04±1.89	72.58±1.36	75.91±2.93	84.41±2.62	67±1.01	60.67±1.9	64.25±0.02	56.18±2.07
	Histidine	0.29±0.12	0.41±0.01	0.59±0.14	0.59±0.20	0.79±0.13	0.86±0.06	0.63±0.05	0.52±0.01
	Isoleucine	1.93±0.01	1.82±0.06	2.21±0.03	1.64±0.06	2.26±0.10	1.91±0.05	1.82±0.03	1.96±0.05

	Leucine	3.26±0.07	2.89±0.04	3.75±0.01	2.73±0.23	3.67±0.27	3.45±0.21	3.22±0.07	3.78±0.12
	Lysine	3.67±0.01	3.30±0.09	3.83±0.32	3.69±0.16	4.74±0.55	3.84±0.30	3.49±0.10	4.24±0.27
	Ornithine	4.15±1.12	4.86±0.42	19.51±6.80	4.24±0.26	5.34±0.77	4.82±0.55	5.74±0.18	8.21±0.04
	Phenylalanine	1.79±0.04	1.55±0.01	2.16±0.11	1.73±0.17	2.41±0.16	1.96±0.23	2.22±0.02	2.31±0.04
	Proline	15.63±0.76	16.76±1.15	16.60±0.39	16.01±0.82	13.80±1.01	13.59±0.06	13.39±0.39	13.88±0.41
	Sarcosine	0.15±0.01	0.15±0.02	0.13±0.01	0.15±0.01	0.15±0.01	0.14±0.01	0.13±0.01	0.12±0.01
	Serine	2.96±0.35	3.28±0.10	0.12±0.02	2.60±0.24	3.29±0.12	2.37±0.10	2.23±0.02	2.26±0.23
	Threonine	3.14±0.03	3.31±0.11	0.98±0.36	2.91±0.04	3.76±0.29	3.85±0.20	3.43±0.25	3.84±0.11
	Tryptophan	0.51±0.02	0.44±0.01	0.52±0.01	0.49±0.01	0.47±0.03	0.41±0.04	0.41±0.01	0.41±0.01
	Tyrosine	1.60±0.12	1.72±0.01	1.31±0.11	1.55±0.10	1.95±0.06	1.60±0.15	1.51±0.06	1.13±0.07
	Methionine	1.42±0.02	1.43±0.01	1.75±0.01	1.54±0.04	2.09±0.12	1.69±0.03	1.83±0.01	1.90±0.03
	Valine	3.35±0.02	3.41±0.07	4.11±0.10	2.95±0.08	3.75±0.16	3.49±0.14	3.28±0.04	3.72±0.27
Carbohydrates met.	Glucose	2.10±0.05	1.68±0.09	0.15±0.04	1.38±0.07	1.11±0.01	1.64±0.02	1.03±0.08	0.41±0.09
Nucleotides	ATP+ADP+AMP	0.84±0.22	0.90±0.01	0.42±0.04	0.57±0.06	0.40±0.02	0.24±0.01	0.24±0.01	0.19±0.01
	Hypoxanthine	1.61±0.33	2.06±0.11	7.48±0.06	1.48±0.02	1.70±0.02	2.18±0.02	1.81±0.03	2.54±0.06
	IMP	3.45±0.46	3.71±0.08	0.10±0.03	4.88±0.33	3.85±0.09	3.24±0.24	3.75±0.06	3.02±0.16
	Inosine	1.20±0.07	1.30±0.01	0.14±0.01	1.28±0.09	0.98±0.03	1.34±0.11	0.89±0.01	0.71±0.02
Organic acids	2-Hydroxybutyrate	-	-	0.09±0.01	-	-	-	-	-
	2-Hydroxyisovalerate	-	-	0.06±0.01	-	-	-	-	-
	2-Oxoglutarate	0.20±0.01	0.16±0.03	0.24±0.01	0.25±0.01	0.17±0.02	0.25±0.03	0.18±0.05	0.15±0.05
	2-Oxoisocaproate	0.05±0.01	0.06±0.01	0.01±0.01	0.05±0.03	0.11±0.01	0.11±0.01	0.06±0.01	0.02±0.01
	3-Methyl-2-oxovalerate	0.06±0.01	0.06±0.01	0.02±0.01	0.08±0.02	0.09±0.01	0.09±0.01	0.06±0.01	0.03±0.01
	Acetate	0.54±0.26	0.65±0.08	22.46±0.08	0.56±0.03	0.59±0.09	1.44±0.13	2.46±0.14	4.05±0.09
	Fumarate	0.11±0.02	0.09±0.01	0.02±0.01	0.09±0.01	0.07±0.01	0.06±0.01	0.06±0.01	0.01±0.01
	Lactate	17.45±0.09	19.64±1.11	13.08±0.27	14.95±1.38	11.22±0.86	13.8±0.36	13.46±0.02	14.77±1.18
	Propionate	0.01±0.01	0.02±0.01	2.51±0.31	0.01±0.01	0.04±0.01	0.06±0.03	0.03±0.01	0.05±0.01
	Pyruvate	0.25±0.04	0.21±0.01	0.08±0.01	0.22±0.10	0.33±0.05	0.41±0.03	0.27±0.03	0.15±0.01
	Succinate	0.20±0.02	0.23±0.01	4.25±0.32	0.23±0.01	0.26±0.01	0.40±0.03	0.38±0.02	0.45±0.02
	Urocanate	0.08±0.01	0.07±0.01	0.11±0.01	0.11±0.01	0.11±0.03	0.13±0.03	0.08±0.01	0.10±0.01
Others	2-Heptanone	-	-	0.14±0.01	-	-	-	-	-
	2-Oxoisovalerate	0.03±0.01	0.05±0.01	-	0.05±0.01	0.06±0.01	0.07±0.01	0.04±0.01	0.02±0.01
	Ethanol	0.57±0.14	0.05±0.01	1.32±0.07	0.31±0.02	0.14±0.01	0.07±0.01	0.11±0.01	1.68±0.16
	Glycerol	0.96±0.22	0.83±0.05	0.12±0.04	1.02±0.08	0.73±0.01	0.95±0.21	0.73±0.01	0.49±0.02

Formate	0.09±0.01	0.08±0.01	1.97±0.54	0.15±0.04	0.10±0.01	0.09±0.03	0.16±0.06	2.93±0.14
Cholate	0.07±0.01	0.06±0.01	0.06±0.01	0.09±0.01	0.06±0.01	0.07±0.02	0.07±0.01	0.06±0.01
Isovalerate	0.03±0.01	0.02±0.01	0.09±0.01	0.03±0.01	0.04±0.01	0.04±0.01	0.06±0.02	0.05±0.01

Table S1, continued. Metabolites of striped prawn samples untreated or treated with HHP of 400, 500 and 600 MPa during chilled storage.

		500 MPa						
		D1	D6	D9	D14	D21	D28	D35
Amines	Cadaverine	-	-	-	-	-	-	-
	Choline	0.41±0.02	0.44±0.01	0.39±0.06	0.34±0.04	0.51±0.08	0.44±0.01	0.43±0.03
	Dimethylamine	0.03±0.01	0.03±0.01	0.02±0.01	0.02±0.01	0.02±0.01	0.02±0.01	0.03±0.01
	O-Phosphocholine	0.24±0.02	0.22±0.01	0.21±0.01	0.22±0.01	0.26±0.01	0.2±0.02	0.23±0.01
	Putrescine	-	-	-	-	-	-	-
	sn-Glycero-3-phosphocholine	0.29±0.03	0.27±0.01	0.29±0.01	0.28±0.03	0.27±0.01	0.33±0.01	0.39±0.01
	Taurine	19.97±0.14	17.83±0.24	19.55±1.3	17.46±0.05	14.48±0.11	16.71±2.64	19.22±0.57
	TMAO	23.12±2.06	20.99±0.42	21.19±0.36	20.01±0.36	19.8±0.62	18.47±1.10	20.87±0.05
	TMA	0.10±0.01	0.09±0.01	0.24±0.04	0.12±0.01	0.13±0.01	0.07±0.01	0.08±0.01
	Trigonelline	0.16±0.01	0.14±0.01	0.18±0.01	0.15±0.01	0.14±0.01	0.13±0.01	0.14±0.01
	Tyramine	-	-	-	-	-	-	-
	Uracil	0.16±0.01	0.14±0.01	0.16±0.01	0.14±0.01	0.14±0.02	0.14±0.03	0.15±0.01
Amino Acids	Alanine	15.11±0.01	14.21±0.27	14.47±0.12	14.77±0.13	19.08±0.26	16.78±0.27	14.59±0.03
	Asparagine	1.86±0.09	1.86±0.02	1.60±0.03	2.27±0.05	1.94±0.13	1.92±0.11	1.99±0.03
	Arginine	12.68±0.11	13.17±0.41	11.6±1.28	13.69±0.21	11.65±0.06	8.97±0.2	9.98±0.66
	Beta-Alanine	0.20±0.01	0.17±0.01	0.18±0.01	0.17±0.01	0.14±0.01	0.15±0.02	0.16±0.01
	Betaine	68.53±0.33	61.65±0.04	56.95±0.89	54.96±0.24	48.91±0.26	50.88±2.57	55.64±0.62
	Creatine	0.12±0.02	0.03±0.01	0.25±0.10	0.06±0.01	0.09±0.01	0.96±0.37	0.05±0.01
	Glutamate	2.43±0.75	3.44±0.37	5.78±2.99	3.70±0.48	3.92±0.01	4.21±0.84	2.91±0.63
	Glutamine	7.03±0.04	6.99±0.09	5.83±0.23	6.51±0.14	4.74±0.03	6.53±0.32	5.71±0.03
	Glycine	76.94±2.3	72.94±2.27	72.14±2.68	62.42±1.59	66.3±0.62	57.54±3.96	68.05±0.34
	Histidine	0.58±0.07	0.69±0.09	0.62±0.05	0.65±0.06	0.80±0.05	0.77±0.10	0.79±0.06
	Isoleucine	1.73±0.05	1.70±0.03	1.72±0.06	2.26±0.12	2.24±0.04	2.12±0.04	1.86±0.11

	Leucine	2.82±0.10	2.81±0.05	2.83±0.11	4.21±0.35	4.18±0.18	3.87±0.30	3.27±0.26
	Lysine	3.26±0.50	3.58±0.20	3.33±0.36	5.19±0.16	5.88±0.15	4.34±0.05	3.94±0.10
	Ornithine	5.58±0.64	4.89±0.44	5.09±0.20	3.99±0.01	5.41±0.21	5.44±0.49	8.09±0.18
	Phenylalanine	1.61±0.10	1.8±0.03	1.71±0.08	2.62±0.05	2.79±0.02	2.38±0.01	2.06±0.11
	Proline	17.32±0.59	16.15±0.18	14.44±0.86	15.64±0.48	10.98±0.22	15.28±0.08	15.61±0.22
	Sarcosine	0.15±0.01	0.14±0.01	0.18±0.01	0.13±0.01	0.13±0.01	0.12±0.01	0.15±0.01
	Serine	2.81±0.18	2.93±0.14	1.84±0.06	3.10±0.13	2.24±0.05	2.33±0.04	1.99±0.01
	Threonine	3.11±0.07	3.47±0.25	2.95±0.04	4.04±0.04	4.37±0.11	3.73±0.14	3.59±0.13
	Tryptophan	0.41±0.04	0.39±0.02	0.41±0.01	0.47±0.02	0.46±0.01	0.42±0.02	0.41±0.05
	Tyrosine	1.42±0.01	1.51±0.07	1.29±0.1	1.84±0.13	1.9±0.04	1.7±0.01	1.37±0.02
	Methionine	1.45±0.06	1.59±0.02	1.52±0.09	2.03±0.06	2.1±0.05	1.85±0.08	1.76±0.01
	Valine	3.07±0.06	3.12±0.03	3.06±0.01	4.07±0.19	3.8±0.01	3.57±0.12	3.44±0.04
Carbohydrates met.	Glucose	1.65±0.14	1.46±0.05	1.31±0.07	1.5±0.07	0.63±0.04	1.59±0.01	1.52±0.03
	ATP+ADP+AMP	0.67±0.06	0.79±0.01	0.51±0.04	0.65±0.02	0.51±0.02	0.43±0.02	0.41±0.01
	Hypoxanthine	1.67±0.17	1.54±0.07	1.66±0.06	1.31±0.01	2.00±0.04	1.53±0.01	2.02±0.06
	IMP	4.19±0.04	4.21±0.10	3.4±0.18	3.21±0.06	3.3±0.02	3.21±0.08	3.05±0.12
	Inosine	1.59±0.01	1.41±0.02	1.66±0.01	1.39±0.04	1.03±0.03	1.51±0.01	1.74±0.04
Organic acids	2-Hydroxybutyrate	-	-	-	-	-	-	-
	2-Hydroxyisovalerate	-	-	-	-	-	-	-
	2-Oxoglutarate	0.18±0.01	0.39±0.18	0.33±0.04	0.32±0.06	0.22±0.01	0.27±0.04	0.3±0.03
	2-Oxoisocaproate	0.02±0.01	0.04±0.01	0.05±0.01	0.04±0.01	0.06±0.01	0.05±0.01	0.04±0.01
	3-Methyl-2-oxovalerate	0.05±0.01	0.06±0.01	0.06±0.01	0.06±0.01	0.05±0.01	0.05±0.01	0.04±0.01
	Acetate	0.49±0.13	0.33±0.02	0.99±0.22	0.51±0.02	0.77±0\.11	0.95±0.15	0.82±0.17
	Fumarate	0.07±0.01	0.07±0.01	0.06±0.01	0.06±0.01	0.04±0.01	0.04±0.01	0.05±0.01
	Lactate	16.41±0.02	14.14±0.21	13.84±0.24	14.4±0.41	7.61±0.76	13.07±0.47	15.51±0.29
	Propionate	0.01±0.01	0.04±0.01	0.04±0.01	0.04±0.01	0.05±0.01	0.03±0.01	0.04±0.01
	Pyruvate	0.24±0.01	0.32±0.05	0.5±0.05	0.39±0.02	0.38±0.01	0.47±0.02	0.45±0.03
	Succinate	0.25±0.01	0.27±0.02	0.32±0.02	0.33±0.02	0.28±0.01	0.45±0.01	0.56±0.04
	Urocanate	0.11±0.03	0.1±0.01	0.06±0	0.05±0.01	0.06±0.01	0.09±0.01	0.06±0.01
Others	2-Heptanone	-	-	-	-	0.02±0.02	-	-
	2-Oxoisovalerate	0.03±0.01	0.03±0.01	0.04±0.01	0.03±0.01	0.03±0.01	0.03±0.01	0.03±0.01
	Ethanol	0.48±0.16	0.07±0.03	0.07±0.01	0.04±0.01	0.06±0.02	0.13±0.01	0.08±0.02
	Glycerol	1.12±0.17	0.77±0.05	6.31±5.23	0.76±0.03	0.68±0.03	0.72±0.01	0.87±0.01

Formate	0.12±0.03	0.09±0.01	0.08±0.01	0.07±0.01	0.17±0.03	0.26±0.01	0.16±0.02
Cholate	0.07±0.01	0.07±0.01	0.07±0.01	0.05±0.01	0.04±0.01	0.07±0.01	0.07±0.01
Isovalerate	0.04±0.01	0.02±0.01	0.03±0.01	0.06±0.01	0.02±0.01	0.03±0.01	0.02±0.01

Table S1, continued. Metabolites of striped prawn samples untreated or treated with HHP of 400, 500 and 600 MPa during chilled storage.

		600 MPa						
		D1	D6	D9	D14	D21	D28	D35
Amines	Cadaverine	-	-	-	-	-	-	-
	Choline	0.42±0.05	0.44±0.03	0.48±0.01	0.38±0.04	0.36±0.01	0.32±0.02	0.33±0.01
	Dimethylamine	0.03±0.01	0.03±0.01	0.03±0.01	0.02±0.01	0.02±0.01	0.02±0.01	0.02±0.01
	O-Phosphocholine	0.24±0.01	0.23±0.02	0.22±0.01	0.27±0.01	0.22±0.01	0.20±0.02	0.17±0.01
	Putrescine	-	-	-	-	-	-	-
	sn-Glycero-3-phosphocholine	0.28±0.03	0.29±0.04	0.25±0.02	0.34±0.02	0.29±0.01	0.29±0.02	0.3±0.02
	Taurine	21.38±0.19	19.56±0.62	16.1±3.45	17.65±0.42	16.14±1.58	14.09±1.13	16.48±0.19
	TMAO	24.82±2.49	21.56±2.00	20.16±0.84	23.09±0.58	15.41±0.81	16.07±1.23	17.34±0.48
	TMA	0.15±0.01	0.09±0.01	0.14±0.01	0.12±0.01	0.1±0.01	0.08±0.01	0.11±0.01
	Trigonelline	0.17±0.02	0.18±0.01	0.12±0.01	0.17±0.01	0.12±0.01	0.12±0.01	0.11±0.01
	Tyramine	-	-	-	-	-	-	-
	Uracil	0.14±0.01	0.15±0.01	0.14±0.02	0.14±0.01	0.14±0.01	0.12±0.02	0.1±0.01
Amino Acids	Alanine	13.47±0.59	14.52±0.94	15.15±0.37	18.17±0.2	14.52±0.5	15±0.09	17.16±0.56
	Asparagine	1.74±0.13	1.93±0.01	1.72±0.01	1.71±0.06	1.86±0.11	2.17±0.07	2.59±0.05
	Arginine	16.22±0.22	14.33±0.33	11.35±1.32	13.17±0.5	11.96±0.41	11.09±0.07	12.7±0.93
	Beta-Alanine	0.19±0.02	0.17±0.01	0.18±0.02	0.17±0.01	0.16±0.01	0.18±0.01	0.16±0.01
	Betaine	75.55±5.96	59.8±2.45	56.27±0.20	57.37±0.12	48.16±0.90	50.06±0.99	50.07±0.27
	Creatine	0.05±0.02	0.04±0.01	0.07±0.02	0.04±0.01	0.08±0.01	0.05±0.01	0.10±0.01
	Glutamate	2.48±1.04	4.17±0.67	3.56±0.95	3.39±0.33	4.00±0.57	6.45±0.72	4.37±1.05
	Glutamine	6.74±0.44	5.60±0.30	5.45±0.05	4.42±0.12	5.34±0.01	5.76±0.02	6.68±0.37
	Glycine	85.26±5.98	73.2±3.86	68.93±2.31	74.92±0.94	55.44±1.08	53.02±1.38	56.81±0.42
	Histidine	0.48±0.14	0.7±0.09	0.56±0.01	0.69±0.15	0.45±0.11	0.72±0.05	0.58±0.04
	Isoleucine	1.60±0.24	1.86±0.09	1.76±0.04	1.73±0.01	2.49±0.04	2.38±0.13	2.73±0.03
	Leucine	2.86±0.39	3.12±0.02	3.17±0.14	2.91±0.05	4.69±0.18	4.58±0.25	5.12±0.20

	Lysine	3.94±1.26	3.98±0.08	3.81±0.13	4.20±0.2	5.55±0.31	5.90±0.05	7.55±0.07
	Ornithine	2.99±0.64	4.05±0.26	5.87±0.07	5.01±0.11	3.77±0.07	4.86±0.02	4.33±0.05
	Phenylalanine	1.69±0.41	1.87±0.09	1.91±0.14	2.08±0.13	2.87±0.15	2.86±0.16	3.55±0.01
	Proline	16.86±2.7	13.61±0.2	12.11±0.38	12.73±0.25	11.39±0.17	12.12±0.53	13.24±0.41
	Sarcosine	0.16±0.01	0.14±0.03	0.15±0.01	0.18±0.01	0.12±0.01	0.13±0.01	0.13±0.01
	Serine	3.44±0.12	3.16±0.01	2.38±0.25	2.57±0.07	3.20±0.28	3.36±0.25	4.06±0.06
	Threonine	2.86±0.10	3.12±0.21	3.2±0.06	3.21±0.26	4.11±0.32	4.27±0.19	4.65±0.20
	Tryptophan	0.43±0.06	0.49±0.04	0.40±0.01	0.45±0.02	0.50±0.02	0.49±0.02	0.55±0.02
	Tyrosine	1.53±0.19	1.78±0.04	1.64±0.01	1.72±0.01	2.17±0.21	2.18±0.05	2.33±0.06
	Methionine	1.53±0.19	1.68±0.05	1.62±0.04	1.82±0.01	2.21±0.08	2.29±0.06	2.56±0.04
	Valine	2.72±0.22	3.17±0.12	2.95±0.09	3.04±0.01	3.83±0.09	3.74±0.10	4.56±0.03
Carbohydrates met.	Glucose	1.50±0.03	1.44±0.05	1.10±0.03	0.97±0.01	1.1±0.05	0.96±0.04	1.34±0.02
	ATP+ADP+AMP	1.30±0.06	1.05±0.01	0.81±0.04	0.7±0.03	0.8±0.01	0.84±0.02	0.78±0.03
	Hypoxanthine	1.17±0.26	1.45±0.05	1.43±0.01	1.70±0.10	1.16±0.01	1.28±0.07	1.24±0.01
	IMP	4.58±0.92	3.95±0.05	3.23±0.19	4.16±0.05	2.4±0.05	2.54±0.04	2.53±0.11
	Inosine	1.58±0.27	1.52±0.07	1.77±0.07	1.60±0.02	1.39±0.01	1.54±0.01	1.61±0.05
Organic acids	2-Hydroxybutyrate	-	-	-	-	-	0.01±0.01	0.01±0.01
	2-Hydroxyisovalerate	-	-	-	-	-	-	-
	2-Oxoglutarate	0.37±0.17	0.27±0.09	0.18±0.04	0.28±0.13	0.28±0.01	0.2±0.02	0.91±0.77
	2-Oxoisocaproate	0.03±0.01	0.03±0.01	0.03±0.01	0.03±0.01	0.04±0.01	0.05±0.01	0.04±0.01
	3-Methyl-2-oxovalerate	0.03±0.01	0.06±0.02	0.07±0.01	0.04±0.01	0.05±0.01	0.06±0.01	0.05±0.01
	Acetate	0.39±0.15	0.4±0.16	0.57±0.09	0.51±0.15	0.61±0.09	0.51±0.13	0.75±0.02
	Fumarate	0.07±0.01	0.07±0.01	0.07±0.01	0.08±0.01	0.05±0.01	0.04±0.01	0.05±0.01
	Lactate	15.54±1.55	12.69±0.43	11.00±0.84	9.97±0.38	11.01±0.01	12.41±0.11	12.29±1.28
	Propionate	0.03±0.01	0.03±0.01	0.03±0.01	0.03±0.01	0.04±0.01	0.04±0.01	0.05±0.01
	Pyruvate	0.09±0.02	0.31±0.01	0.60±0.01	0.42±0.07	0.34±0.01	0.46±0.01	0.42±0.06
	Succinate	0.23±0.01	0.23±0.01	0.27±0.01	0.26±0.01	0.31±0.01	0.32±0.01	0.39±0.02
	Urocanate	0.05±0.01	0.05±0	0.08±0.01	0.08±0.01	0.06±0.01	0.07±0.01	0.06±0.01
Others	2-Heptanone	-	-	-	-	-	-	-
	2-Oxoisovalerate	0.02±0.01	0.03±0.01	0.03±0.01	0.03±0.01	0.02±0.01	0.03±0.01	0.02±0.01
	Ethanol	0.22±0.03	0.04±0.01	0.06±0.02	0.05±0.01	0.06±0	0.08±0.03	0.07±0.01
	Glycerol	1.01±0.04	0.86±0.03	0.91±0.13	0.7±0.02	0.82±0.02	0.77±0.02	0.99±0.21
	Formate	0.10±0.02	0.08±0.01	0.08±0.02	0.09±0.01	0.08±0.01	0.08±0.01	0.08±0.01

Cholate	0.08±0.02	0.06±0.01	0.06±0.01	0.06±0.01	0.06±0.01	0.06±0.01	0.05±0.01
Isovalerate	0.03±0.01	0.04±0.01	0.04±0.01	0.05±0.01	2.13±2.11	0.06±0.03	0.07±0.04

Table S2. Taste, taste threshold, and TAVs of taste active molecules in untreated and treated (400, 500, 600 MPa) striped prawns during storage.

Taste		Sour			Sweet						
Molecule		lactate	succinate	acetate	Glycine	Alanine	Lysine	Serine	Proline	Threonine	Glucose
Threshold (mg/g)		1.3[a]	0.1[a]	0.12[a]	1.3[b]	0.6[b]	0.5[b]	1.5[b]	3[b]	2.6[b]	0.86[a]
Control	Day1	**1.19±0.03**	0.23±0.02	0.26±0.17	**3.69±0.08**	**1.85±0.05**	**1.05±0.02**	0.20±0.04	0.59±0.05	0.14±0.01	0.43±0.01
	Day6	**1.35±0.10**	0.27±0.02	0.32±0.06	**4.16±0.14**	**1.84±0.04**	0.96±0.03	0.23±0.01	0.64±0.07	0.15±0.01	0.35±0.03
	Day9	0.88±0.02	**4.87±0.47**	**10.91±0.18**	**4.25±0.18**	**2.43±0.01**	**1.09±0.14**	0.01±0.01	0.62±0.01	0.04±0.02	0.03±0.01
400 MPa	Day1	**1.01±0.07**	0.27±0.04	0.27±0.01	**4.66±0.08**	**2.03±0.08**	**1.04±0.13**	0.17±0.01	0.59±0.01	0.13±0.01	0.28±0.04
	Day6	0.77±0.08	0.30±0.01	0.29±0.06	**3.84±0.09**	**2.38±0.07**	**1.38±0.22**	0.23±0.01	0.53±0.05	0.17±0.02	0.23±0.01
	Day9	0.96±0.02	0.47±0.04	0.72±0.09	**3.51±0.20**	**2.3±0.16**	**1.12±0.14**	0.17±0.01	0.52±0.01	0.18±0.02	0.34±0.01
	Day14	0.93±0.01	0.44±0.04	**1.23±0.10**	**3.69±0.01**	**2.83±1.01**	**0.67±0.45**	0.08±0.11	0.67±0.20	0.09±0.11	0.22±0.02
	Day21	**1.00±0.11**	0.52±0.03	**1.97±0.06**	**3.16±0.17**	**2.32±0.10**	**1.21±0.11**	0.15±0.02	0.52±0.02	0.17±0.01	0.08±0.03
500 MPa	Day1	**1.12±0.02**	0.29±0.01	0.24±0.09	**4.39±0.26**	**2.22±0.04**	0.94±0.19	0.19±0.01	0.66±0.04	0.14±0.01	0.34±0.03
	Day6	**1.01±0.02**	0.33±0.03	0.17±0.02	**4.36±0.21**	**2.19±0.07**	**1.08±0.08**	0.21±0.01	0.64±0.01	0.16±0.02	0.32±0.02
	Day9	0.95±0.02	0.38±0.04	0.49±0.16	**4.14±0.18**	**2.14±0.04**	0.97±0.14	0.13±0.01	0.55±0.04	0.13±0.01	0.27±0.02
	Day14	0.99±0.04	0.38±0.03	0.25±0.01	**3.56±0.14**	**2.17±0.02**	**1.50±0.06**	0.21±0.01	0.59±0.02	0.18±0.01	0.31±0.02
	Day21	0.94±0.06	0.34±0.02	0.40±0.07	**3.97±0.17**	**2.93±0.03**	**1.78±0.01**	0.16±0.01	0.44±0.02	0.21±0.01	0.14±0.02
	Day28	0.90±0.04	0.53±0.01	0.48±0.10	**3.32±0.35**	**2.49±0.08**	**1.27±0.03**	0.16±0.01	0.59±0.01	0.17±0.01	0.33±0.01
	Day35	**1.11±0.02**	0.68±0.08	0.42±0.13	**4.04±0.01**	**2.23±0.03**	**1.19±0.06**	0.14±0.01	0.62±0.02	0.17±0.01	0.33±0.01
600 MPa	Day1	**1.15±0.02**	0.27±0.01	0.19±0.11	**4.89±0.46**	**1.99±0.11**	**1.14±0.52**	0.24±0.01	0.64±0.14	0.13±0.01	0.31±0.01
	Day6	0.88±0.04	0.28±0.02	0.20±0.11	**4.22±0.31**	**2.15±0.20**	**1.16±0.03**	0.22±0.01	0.52±0.01	0.14±0.01	0.30±0.02
	Day9	0.77±0.08	0.32±0.01	0.29±0.07	**4.01±0.17**	**2.27±0.09**	**1.12±0.05**	0.17±0.02	0.47±0.02	0.15±0.01	0.23±0.01
	Day14	0.69±0.03	0.30±0.01	0.25±0.11	**4.31±0.10**	**2.69±0.06**	**1.22±0.09**	0.18±0.01	0.49±0.02	0.15±0.02	0.20±0.01
	Day21	0.79±0.01	0.38±0.01	0.32±0.06	**3.33±0.09**	**2.24±0.11**	**1.69±0.13**	0.23±0.03	0.45±0.01	0.20±0.02	0.24±0.02
	Day28	0.89±0.03	0.39±0.01	0.26±0.09	**3.17±0.05**	**2.31±0.07**	**1.79±0.06**	0.24±0.02	0.48±0.02	0.20±0.02	0.21±0.02
	Day35	0.87±0.10	0.47±0.01	0.39±0.03	**3.38±0.15**	**2.62±0.21**	**2.27±0.05**	0.29±0.02	0.52±0.01	0.22±0.01	0.29±0.01

Table S2, continued. Taste, taste threshold, and TAVs of taste active molecules in untreated and treated (400, 500, 600 MPa) striped prawns during storage.

Taste		Umami		Bitter								
Molecule		IMP	Glutamate	Phenylalanine	Tyrosine	Histamine	Arginine	Methionine	Isoleucine	Valine	Leucine	Tryptophan
Threshold (mg/g)		0.25[c]	0.3[b]	0.9[b]	0.91[a]	0.2[b]	0.5[b]	0.3[b]	0.9[b]	0.4[b]	1.9[b]	0.9[a]
control	Day1	**4.71±0.94**	0.82±0.01	0.32±0.02	0.31±0.04	0.22±0.13	**4.92±0.03**[*]	0.70±0.03	0.28±0.01	0.96±0.01	0.22±0.01	0.11±0.01
	Day6	**5.01±0.21**	0.65±0.09	0.28±0.01	0.34±0.01	0.32±0.01	**4.13±0.40**	0.71±0.01	0.26±0.01	0.99±0.04	0.20±0.01	0.10±0.01
	Day9	0.14±0.06	0.57±0.08	0.38±0.03	0.25±0.03	0.44±0.15	**2.03±0.56**	0.84±0.02	0.31±0.01	**1.17±0.03**	0.25±0.01	0.11±0.01
400 MPa	Day1	**6.44±0.19**	0.70±0.22	0.31±0.06	0.30±0.04	0.45±0.23	**4.50±0.12**	0.73±0.07	0.23±0.03	0.83±0.08	0.18±0.03	0.11±0.01
	Day6	**5.34±0.15**	0.56±0.09	0.44±0.04	0.39±0.02	0.60±0.13	**3.59±0.36**	**1.03±0.08**	0.33±0.02	**1.09±0.06**	0.25±0.03	0.11±0.01
	Day9	**4.15±0.03**	0.61±0.10	0.36±0.07	0.32±0.05	0.66±0.07	**4.10±0.13**	0.84±0.03	0.28±0.01	**1.02±0.07**	0.24±0.02	0.09±0.01
	Day14	**5.23±0.10**	0.37±0.04	0.24±0.23	0.17±0.20	0.27±0.36	**2.57±1.07**	0.53±0.53	0.16±0.15	0.67±0.39	0.15±0.10	0.05±0.06
	Day21	**4.27±0.24**	0.33±0.02	0.41±0.01	0.22±0.02	0.39±0.01	**3.14±0.58**	0.92±0.03	0.28±0.01	**1.06±0.11**	0.25±0.01	0.09±0.01
500 MPa	Day1	**5.74±0.01**	0.43±0.16	0.29±0.02	0.28±0.01	0.45±0.08	**4.36±0.13**	0.71±0.03	0.25±0.01	0.89±0.01	0.19±0.01	0.09±0.01
	Day6	**5.92±0.15**	0.46±0.04	0.34±0.01	0.31±0.02	0.56±0.10	**4.75±0.19**	0.82±0.01	0.26±0.01	0.95±0.01	0.20±0.01	0.09±0.01
	Day9	**4.71±0.32**	0.55±0.01	0.31±0.02	0.26±0.03	0.48±0.06	**4.51±0.11**	0.75±0.05	0.25±0.01	0.89±0.01	0.19±0.01	0.09±0.01
	Day14	**4.46±0.08**	0.60±0.14	0.47±0.01	0.36±0.04	0.49±0.06	**4.71±0.12**	**1.00±0.04**	0.33±0.02	**1.18±0.07**	0.29±0.03	0.11±0.01
	Day21	**4.86±0.07**	0.37±0.12	0.53±0.01	0.39±0.02	0.64±0.04	**4.20±0.15**	**1.08±0.01**	0.34±0.01	**1.15±0.04**	0.30±0.01	0.11±0.01
	Day28	**4.51±0.20**	0.49±0.10	0.44±0.01	0.34±0.01	0.59±0.10	**3.12±0.07**	0.92±0.06	0.31±0.01	**1.05±0.06**	0.27±0.03	0.09±0.01
	Day35	**4.34±0.31**	0.44±0.13	0.39±0.02	0.28±0.01	0.63±0.08	**3.57±0.30**	0.90±0.01	0.28±0.03	**1.04±0.03**	0.23±0.02	0.10±0.02
600 MPa	Day1	**7.34±0.06**	0.65±0.05	0.31±0.11	0.30±0.06	0.37±0.15	**5.61±0.07**	0.75±0.14	0.23±0.05	0.79±0.10	0.20±0.04	0.10±0.02
	Day6	**5.51±0.13**	0.38±0.06	0.34±0.02	0.35±0.01	0.54±0.10	**4.98±0.16**	0.83±0.04	0.27±0.02	0.93±0.05	0.22±0.01	0.11±0.01
	Day9	**4.53±0.36**	0.29±0.16	0.35±0.03	0.33±0.01	0.44±0.01	**4.48±0.08**	0.81±0.02	0.26±0.01	0.87±0.03	0.22±0.01	0.09±0.01
	Day14	**5.75±0.16**	0.38±0.01	0.38±0.04	0.34±0.01	0.63±0.03	**4.57±0.22**	0.90±0.01	0.25±0.01	0.89±0.01	0.20±0.01	0.10±0.01
	Day21	**3.39±0.17**	0.38±0.03	0.55±0.04	0.45±0.06	0.36±0.12	**4.33±0.20**	**1.14±0.06**	0.38±0.01	**1.17±0.04**	0.34±0.02	0.12±0.01
	Day28	**3.62±0.04**	0.34±0.21	0.54±0.05	0.45±0.02	0.58±0.07	**4.00±0.12**	**1.18±0.06**	0.36±0.03	**1.13±0.07**	0.33±0.02	0.12±0.01
	Day35	**3.49±0.45**	0.51±0.07	0.67±0.03	0.48±0.03	0.46±0.07	**4.55±0.31**	**1.31±0.01**	0.41±0.01	**1.37±0.04**	0.36±0.03	0.13±0.01

[a] Taste threshold value (mg/g) in water was obtained from Rotzoll *et al.*,2006 (Taste preferences in fishes, Taste of amino acids; Quantitative Studies, Taste Reconstitution, and Omission Experiments on the Key Taste Compounds in Morel Mushrooms (Morchella deliciosa Fr.))

[b] Taste threshold value (mg/mL) of free amino acids in water was obtained from the literature: Kato *et al*., 1989 (Role of free amino acids and peptides in food taste)

[c] Taste threshold value (mg/mL) of 5- nucleotides in water was obtained from the literature: Yamaguchi *et al*., 1971 (Measurement of the relative taste intensity of some α-amino acid and 5′-nucleotides)

[*] TAV values higher than 1 are highlighted by bold font.
